# Deltacron is a recombinant variant of SARS-CoV-2 but not a laboratory mistake

**DOI:** 10.1016/j.amsu.2022.104032

**Published:** 2022-06-20

**Authors:** Mohsen Karbalaei, Masoud Keikha

**Affiliations:** Department of Microbiology and Virology, School of Medicine, Jiroft University of Medical Sciences, Jiroft, Iran; Department of Microbiology and Virology, Faculty of Medicine, Mashhad University of Medical Sciences, Mashhad, Iran

Dear Editor;

Recently, Professor Leondios Kostrikis submitted a 25 nucleotide sequence of the new SARS-CoV-2 variant to the Global Initiative for Sharing All Influenza Data (GISAID) database. Moreover, researchers at the University of Cyprus in Nicosia also submitted another 27 nucleotide sequence called “Deltacron” a few days later. However, some scientists pointed to contamination in the laboratory, but other researchers believed that there is a laboratory error in reducing the binding of primers due to mutations in the spike protein gene [1]. Therefore, Krutika Kuppalli tweeted that there is no Deltacron on 9 January.

In general, new variants of SARS-CoV-2 are identified by several types of mutations such as insertions, deletions, variations, as well as genomic recombination [2]. Before the emergence of Deltacron, all five previous variants (Alpha, Beta, Gamma, Delta, and Omicron) of SARS-CoV-2 were being generated due to point mutations in the virus genome, but the new variant, Deltacron, is considered to be the first recombinant variant of SARS-CoV-2 [3]. Development of a new strain requires to coinfection by two SARS-CoV-2 variants in the same host depending on the duration of their co-circulation [4]. In this regard, Rockett et al. reported coinfection with Omicron and Delta variants of SARS-CoV-2 in two separate patients with chronic kidney disease [5]. The first case of Deltacron was reported rom Cyprus on the January 7, 2022 [1]. In recent months, Deltacron has been identified from France, the United States, the United Kingdom, the Netherlands, and Denmark (https://www.livescience.com/deltacron-variant-confirmed). Moreover, the United Kingdom Health Security Agency (UKHSA) has reported 30 cases of Deltacron in the United Kingdom (https://www.theguardian.com/world/2022/mar/11/what-is-deltacron-covid-variant-uk). Deltacron comprise a backbone of Delta variant as well as Omicron spike protein (Omicron-spike/Delta-backbone; the new virus is able to induce both Delta variant (large syncytia cell) and Omicron variant (cell rounding/detachment) features [4,6].

According to recent findings, among previous variants of SARS-CoV-2, the Delta variant causes more severe disease, whereas, Omicron is more contagious than Delta (and less virulent), hence, the recombination of these variants can lead to the emergence of a more virulent variant; in the new variant (Deltacron), the N-terminal domain (NTD) of the spike protein becomes larger, flatter, and more electropositive, which in turn facilitates virus binding to the host cell and ultimately further infectivity [4]. The recombination phenomenon can accelerate viral evolution and may lead to selective advantages such as increased transmission or immune escape [7].

Duerr et al. reported an infection with Deltacron in an immunosuppressed patient treated with Sotrovimab. The results of whole genome sequencing (WGS) of the new variant showed a recombination in its spike N-terminal domain (a 5′ Delta AY.45 portion and a 3′ Omicron BA.1 portion); In this study, they hypothesized that the use of antiviral monoclonal antibodies in immunocompromised individuals could not effectively eradicate the virus, which in turn provided an opportunity for the emergence of new variants of SARS-CoV-2 in mixed-infections [6]. After identifying Omicron in different parts of Africa, Gao et al. announced that Omicron may have evolved within immunocompromised community with a poorer vaccination rate in South Africa [8]. The major part of the recombination in Deltacron is related to the N-terminal domain of the spike gene, which consists of the receptor-binding domain (RBD) of the Omicron variant plus the non-structural ORF1a/b genes of the delta variant [9].

In their study, Starr et al. suggested that the use of Sotrovimab by Covid-19 patients potentially induced selective pressure, which in turn may lead to the emergence of variants with the E340D mutation; this mutation reduces the neutralizing effect of Sotrovimab by 10-fold, thus, antiviral monoclonal antibodies should be considered as a stimulating factor for recombination and the emergence of more resistant variants [10].

There are 34 mutations in Deltacron variant containing ORF1a, S, M, ORF7a, ORF7b, and N that have 27 unique mutations. There are concerns about the ability of this variant to escape the immune system and resist neutralizing antibodies. However, based on the early reports, it seems that not only the prevalence of Omicron is rare, but also the severe clinical outcomes of this variant are rare.

In conclusion, the Deltacron is a new SARS-CoV-2 variant that is currently under investigation. Deltacron is actually a recombinant Delta virus containing the Omicron spike protein. Although there is concern about the infectivity as well as the severity of the disease, there is limited information about this variant. Consumption of antiviral monoclonal antibodies by immunosuppressed individuals may lead to the potential selection between different variants of SARS-CoV-2 and eventually the emergence of new recombinant variants. So, vaccination of these individuals is considered as the best strategy against the emergence and spread of new variants. South Africa has a proportion of HIV-infected people than other countries; lack of timely identification and treatment of HIV immune-compromised individuals provides an appropriate opportunity for the replication of SARS-CoV-2, and the emergence of new variants. Although Deltacron infection was not severe in recent case report studies, there is limited information on the severity of this type of infection. Due to multiple Deltacron polymorphisms, conventional PCR methods are not able to detect the virus, so today the WGS is the only strategy to detect Deltacron. Since the WGS method is not common in the laboratories of developing countries, therefore, the characteristics of this variant may be underestimated. Finally, more research needs to be done on the infection and severity of this virus, as well as the effectiveness of current vaccines.

## Ethical approval

Not applicable for this study.

## Sources of funding

There is no fund for this manuscript.

## Author contribution

Masoud Keikha contribue in conceptional, study design, review of the literatures, writing the draft and revision. Mohsen Karbalaei contribute in critical revision and wrote manuscript.

## Registration unique identifying number (UIN)

Name of the registry: Not applicable.

Unique Identifying number or registration ID: Not applicable.

Hyperlink to your specific registration (must be publicly accessible and will be checked): Not applicable.

## Guarantor

Not applicable for this study.

## Declaration of competing interest

There is no conflict of interest.
